# Supplementary material for: Time or distance encoding by hippocampal neurons via heterogeneous ramping rates
Source: Nat Commun. 2025 Dec 17;16:11083. doi: 10.1038/s41467-025-67038-3 (PMC12712063; doi:10.1038/s41467-025-67038-3)
Supplement: Supplementary file 1 — Supplementary Information [file 41467_2025_67038_MOESM1_ESM.pdf]

Supplementary Fig. 1

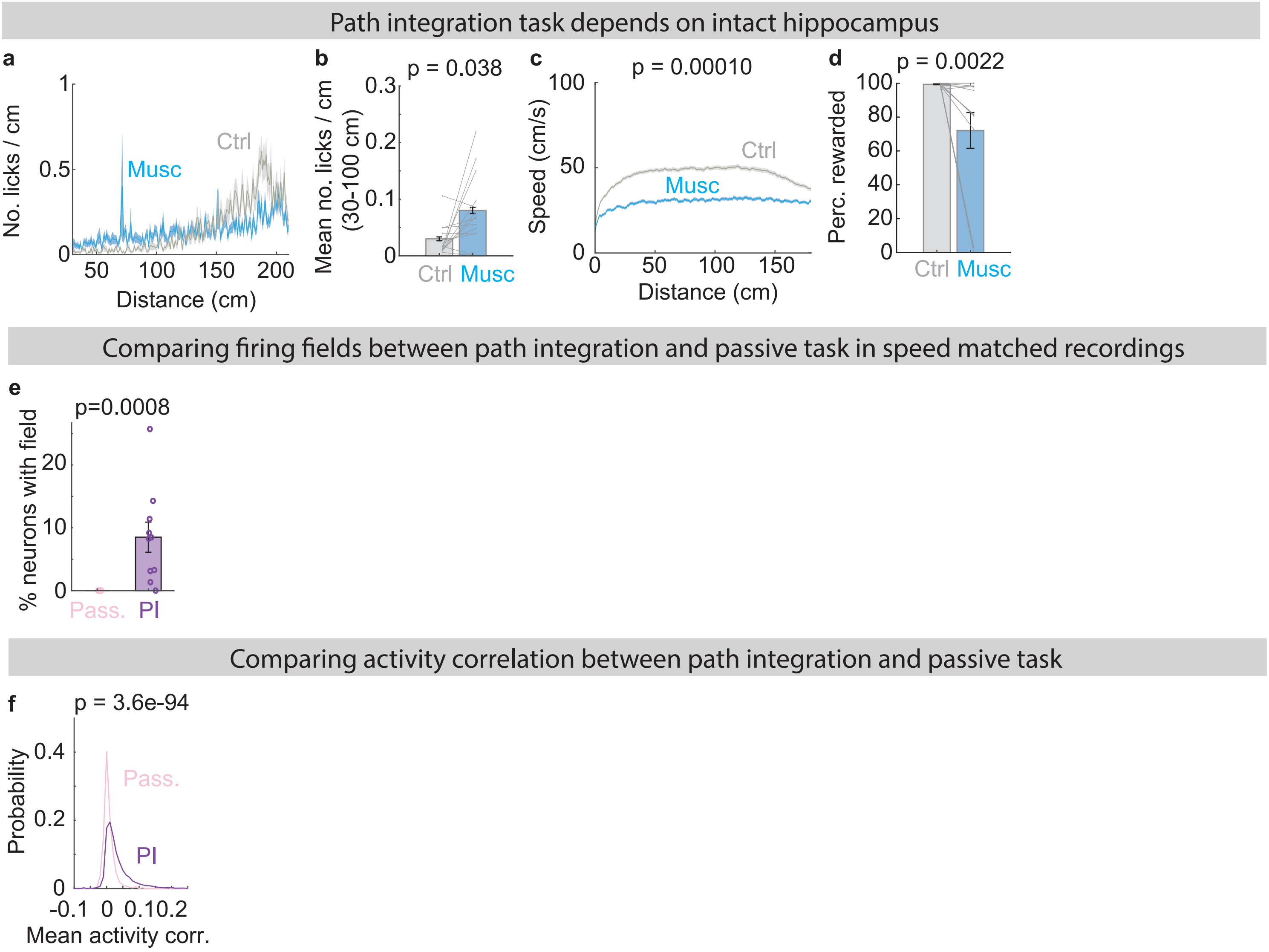

Supplementary Fig. 1: Firing fields in the hippocampal-dependent PI task and the passive task

(a-d) The hippocampal dependence of the PI task is assessed by muscimol infusion experiments targeting CA1. (a) Mean lick histogram binned by distance during the control (grey) and muscimol (blue) sessions (9 animals, 10 recordings). (b) Mean number of licks/cm in the early portion of the trial. (c) Average speed curves over distance. Reported p-value for trial mean speed. (d) The percentage of trials in which the animal received a reward.

(e) Percentage of firing fields in the passive and PI tasks, calculated on PI recordings that matched the mean speed of passive recordings as shown in Fig. 1d.

(f) Distribution of mean trial-by-trial activity correlations of all the pyramidal neurons recorded in the passive and PI tasks.

Supplementary Fig. 2

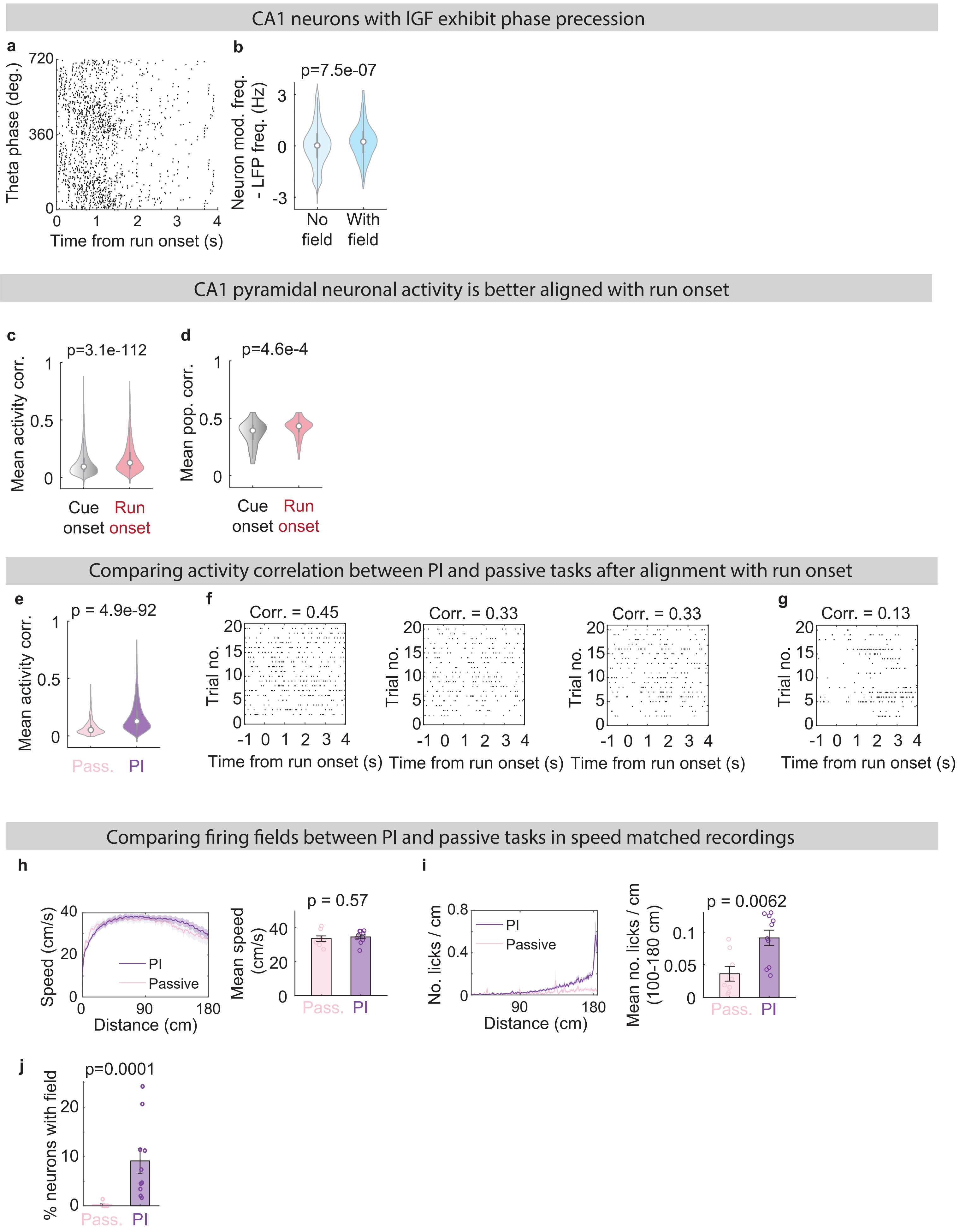

Supplementary Fig. 2: CA1 neuronal activity after run onset alignment

(a-b) Phase precession in neurons with IGFs. (a) A raster plot showing the theta phase change over time for the example pyramidal neuron in Fig. 1f right that has an IGF and demonstrates phase precession. The spikes are aligned with run onset. (b) Pyramidal neurons with fields display significant phase precession relative to those without fields after aligning with run onset (7178 neurons). Phase precession is calculated as the difference between the oscillating frequency of individual neurons and the local field potential theta oscillation frequency.

(c-d) Alignment of all pyramidal neurons. (c) Mean activity correlation aligned with cue vs. run onset for all pyramidal neurons. (d) Mean population correlation aligned with cue vs. run onset for all pyramidal neurons per recording (102 recordings).

(e) Violin plot of trial-by-trial activity correlations of all the pyramidal neurons recorded in the passive and PI tasks after run-onset alignment.

(f) Spike rasters of three example neurons with the highest trial-by-trial activity correlation in the passive task after run-onset alignment. These neurons did not exhibit firing fields, suggesting that activity correlation alone does not predict field presence.

(g) One neuron exhibited a firing field after run-onset alignment in the passive task.

(h-i) Averaged speed traces (h left), mean running speed (h right), lick histograms (i left), and mean number of licks/cm between 100-180 cm during the cue-constant segment (i right) across mice trained in either the passive task or PI task, calculated after run onset alignment. Same speed-matched recordings as in Fig. 1d.

(j) Percentage of firing fields in the passive and PI tasks after run onset alignment, calculated on speed-matched recordings as shown in Fig. 1d.

Supplementary Fig. 3

GLM analyses on neurons with IGFs

a **T(11.7%) D(14.3%) TD(42.1%) p(0.13)**

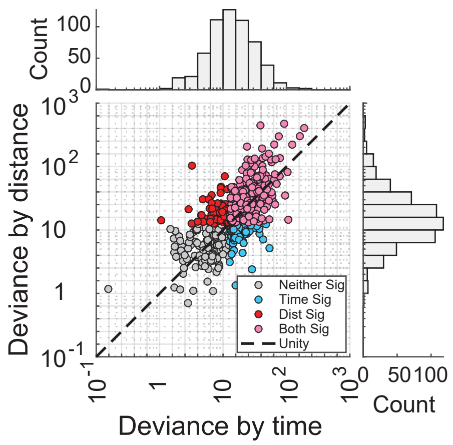

Supplementary Fig. 3: GLM analyses on neurons with IGF

(a) Time versus distance contribution to IGF activity. X-values represent the deviance explained by time (see Methods). Y-values represent the deviance explained by distance. Each point represents a single neuron. Points colored in pink are significantly influenced by both time and distance. Points colored in cyan are significantly influenced by just time. Points colored in red are significantly influenced by just distance. Points colored in grey are not significantly influenced by either time or distance. The top panel shows the histogram of deviance by time, and the right panel shows the histogram of deviance by distance. Title shows the percentage of neurons that are significantly influenced by just time (T), just distance (D), and both time and distance (TD). P-value is calculated using Wilcoxon rank-sum test between the deviance by time and by distance.

Supplementary Fig. 4

Theta oscillations remain during the brief stop before run onset

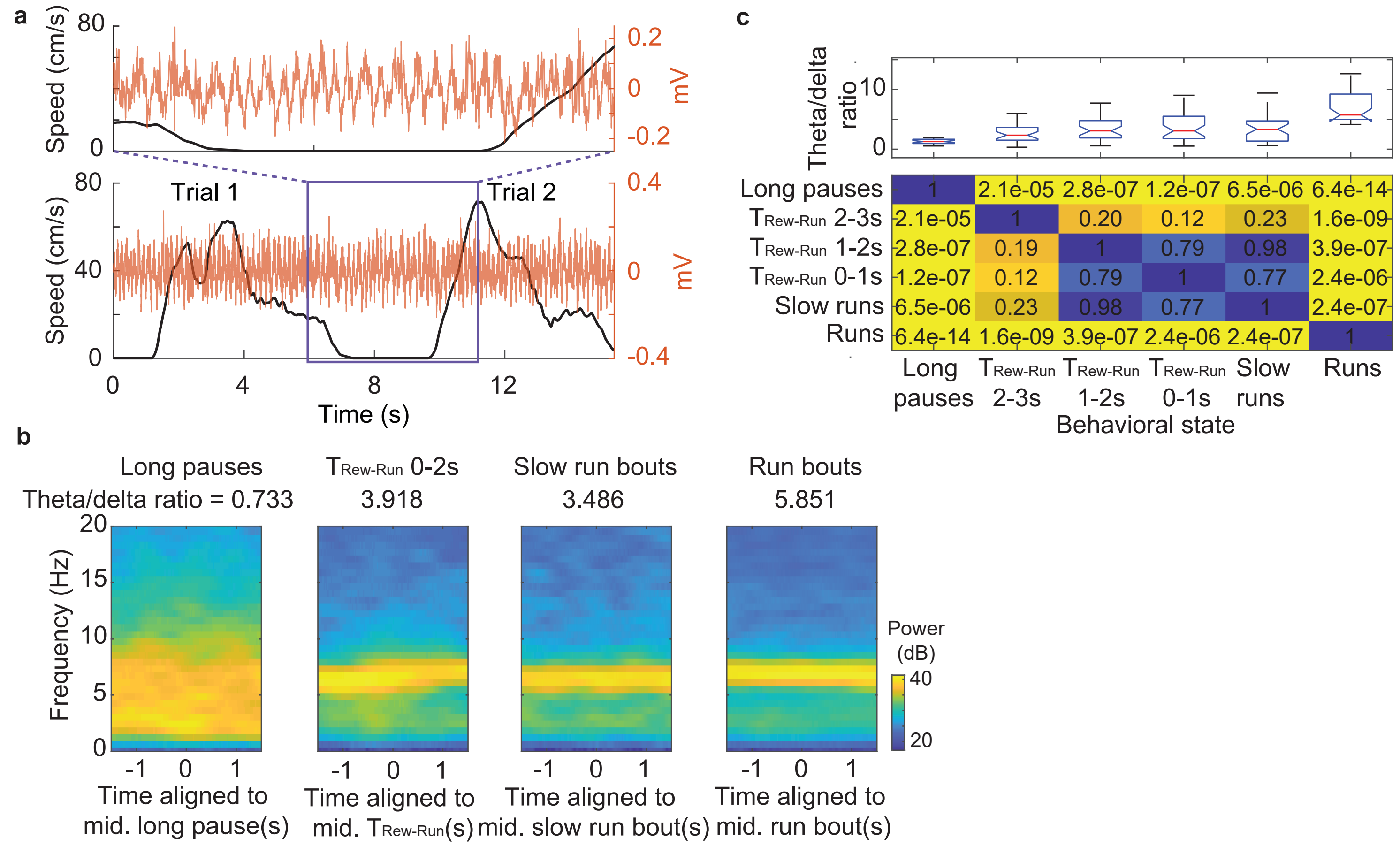

Supplementary Fig. 4: Theta oscillations remain during short stops between trials

(a-c) Theta oscillations are unchanged when an animal initiates running at the beginning of each trial after stopping to consume reward at the end of the last trial. (a) An example showing that theta oscillations persist during the brief stop before run onset. Black: running speed. Orange: raw local field potential trace. Top: zooming in on the segment inside the purple box. (b) Averaged multitaper spectrograms and theta/delta ratios aligned with the midpoints of long pauses (left), trials with a $T_{Rew\text{-}Run}$ between 0-2s, calculated as the time between the last trial's reward delivery and the current trial's run onset (middle left), slow running (speed 5-20 cm/s, middle right) or running bouts (right) in one example recording. (c) Top: distribution of theta/delta ratios for different behavioral states pooled across recordings (13 animals, 25 recordings). Bottom: p-value matrix comparing theta/delta ratios across behavioral states using pairwise Kruskal-Wallis tests. There is no significant change during the pause between trials and slow running (speed 5-20 cm/s).

Supplementary Fig. 5

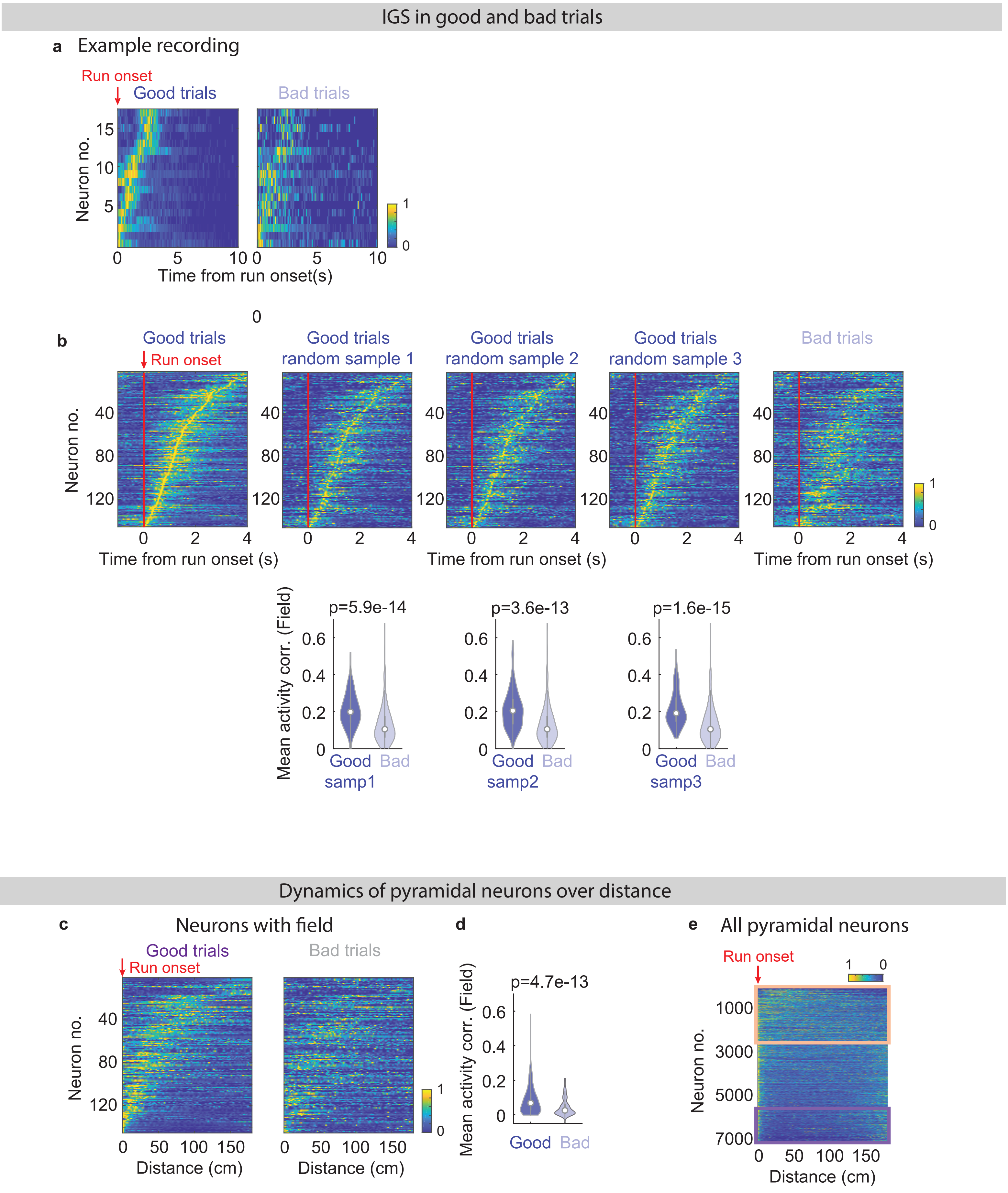

Supplementary Fig. 5: IGS over time or distance compared across good and bad trials

(a) An example IGS from the same session as in Fig. 2f averaged across good (left) and bad (right) trials.

(b) Random subsampling of good trials to match the number of bad trials. Top row: left, IGS from good trials, the same as Fig. 3c left; panels 2-4: IGS from three iterations of subsampled good trials; right, IGS from bad trials, the same as Fig. 3c right. Bottom row: mean trial-by-trial activity correlation of neurons with IGFs, broken down by subsampled good (blue) and bad trials (light blue).

(c) IGS, the same as in Fig. 3c, plotted over distance.

(d) Mean trial-by-trial activity correlation of neurons with IGFs, broken down by good (blue) and bad trials (light blue). Same as in Fig. 3d, but the correlation is calculated based on the firing rate profiles over distance.

(e) Normalized firing rate heatmaps of all pyramidal neurons ordered by their firing rate ratio R. Same as in Fig. 3e, plotted over distance.

Supplementary Fig. 6

IGS in good and bad trials (without no stop criterion for bad trials)

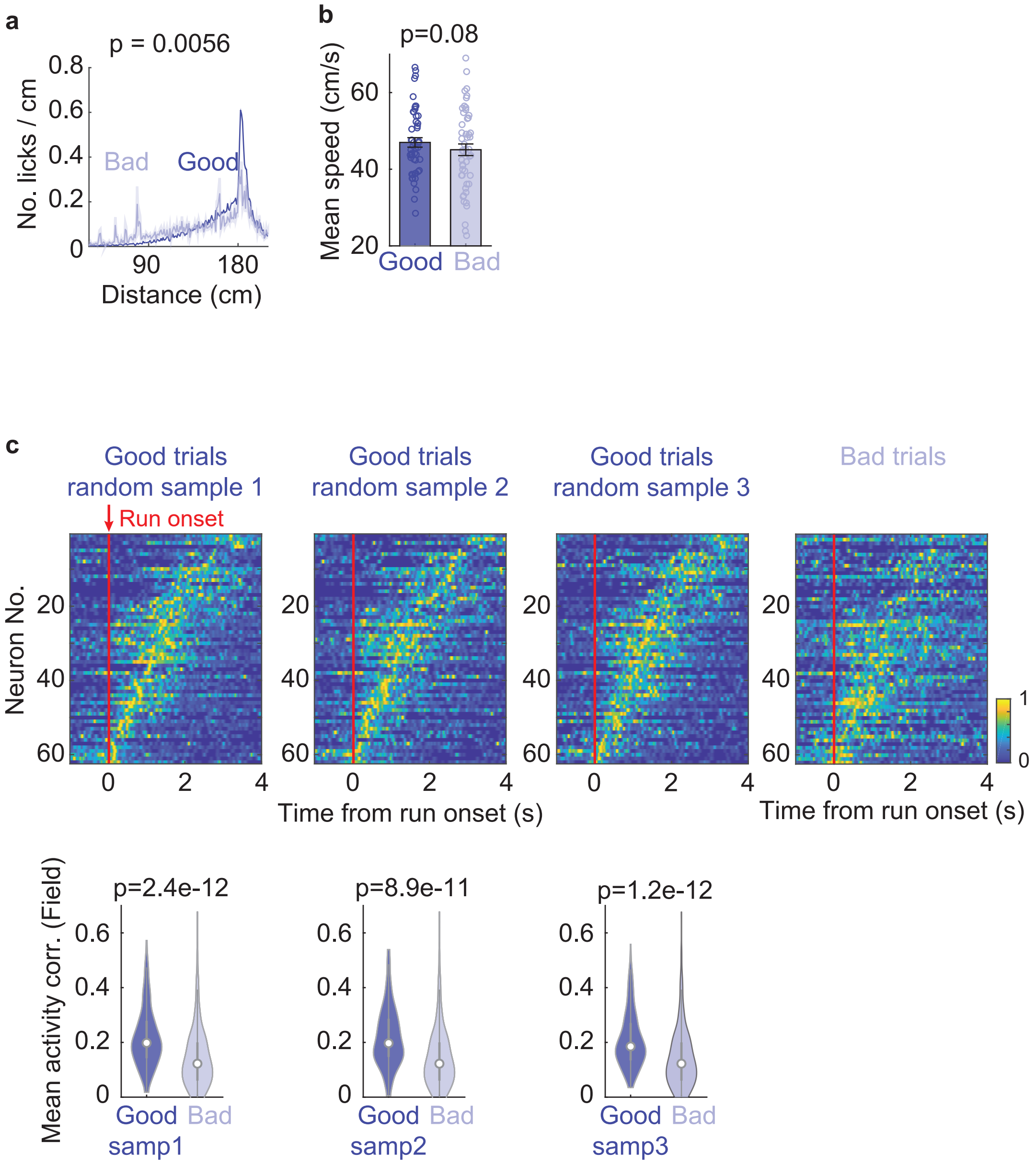

Supplementary Fig. 6: IGS over time or distance compared across good and bad trials, after adding complete stop criterion to bad trials

(a-b) Lick histograms (reported p-value is for mean number of licks/cm between 30-100 cm) (a) and mean running speed (b), broken down by good (blue) and bad trials (light blue) (10 animals, 13 recordings, 1142 good trials, 339 bad trials).

(b) Similar to Supplementary Fig. 5b, for the new good and bad trial definition.

Supplementary Fig. 7

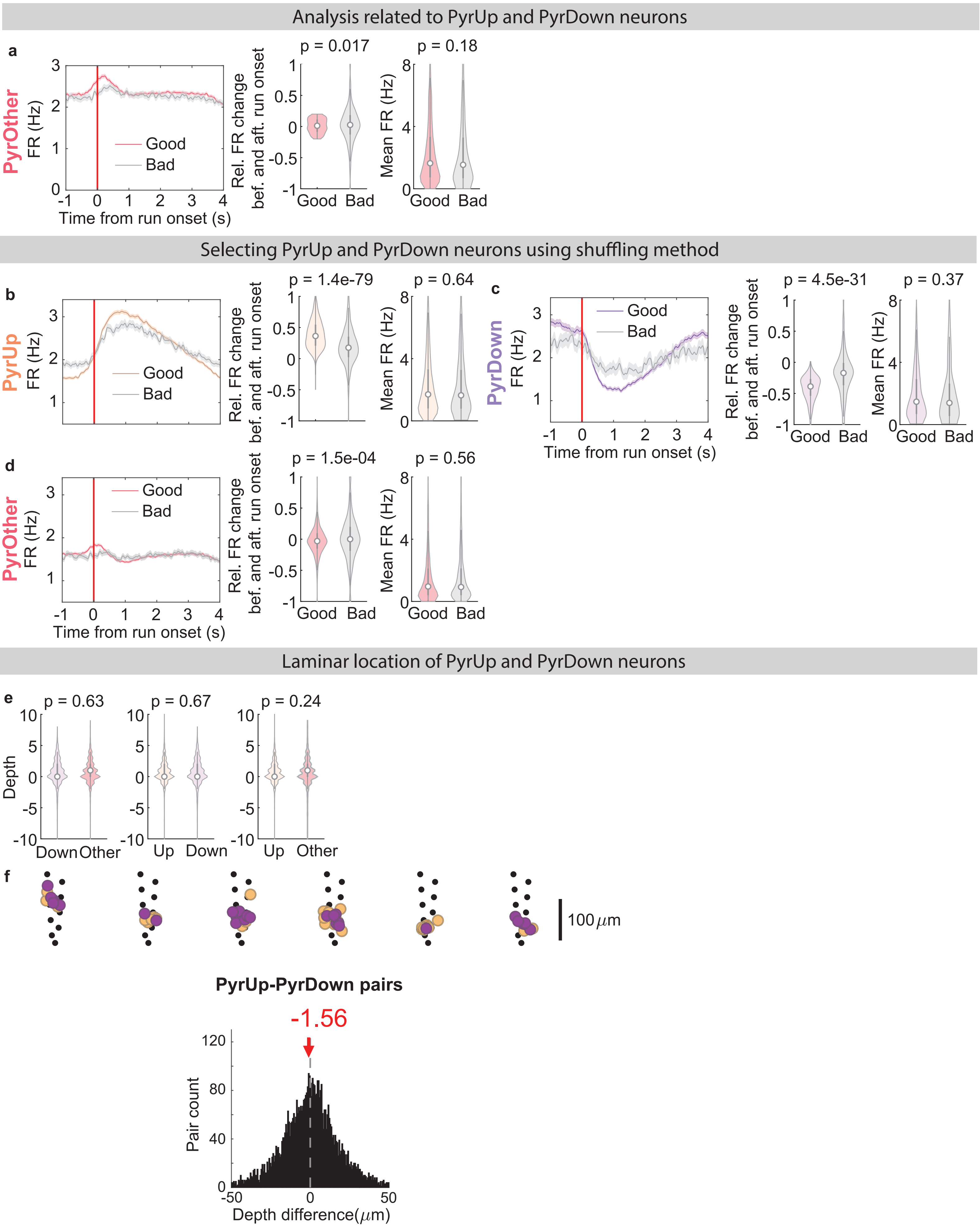

Supplementary Fig. 7: Pyramidal neurons display distinct responses around run onset

(a) Firing rate profiles averaged across all PyrOther neurons (2998 neurons), broken down into good and bad trials (Left). Relative firing rate change around run onset (Middle), and overall mean firing rate calculated between -1s and 4s (Right).

(b) Firing rate profiles for PyrUp neurons identified by the shuffling method (2943 neurons, see Methods) in good and bad trials (left). Relative firing rate change around run onset (middle) and overall mean firing rate (right).

(c) Same as in (b), for PyrDown neurons identified by the shuffling method (923 neurons).

(d) Same as in (b), for PyrOther neurons identified by the shuffling method (3154 neurons).

(e) Depth distribution comparing all PyrDown and PyrOther neurons (left), PyrUp and PyrDown neurons (middle), and PyrUp and PyrOther neurons (right).

(f) Top: example of PyrUp (yellow) and PyrDown (purple) neurons' location along the six shanks of a silicon probe with overlaid silicon probe geometry. Bottom: distribution of depth differences between PyrUp-PyrDown neuron pairs from the same shank[25] (see Methods). Red: mean depth difference.

Supplementary Fig. 8

GLM analyses for PyrUp and PyrDown neurons

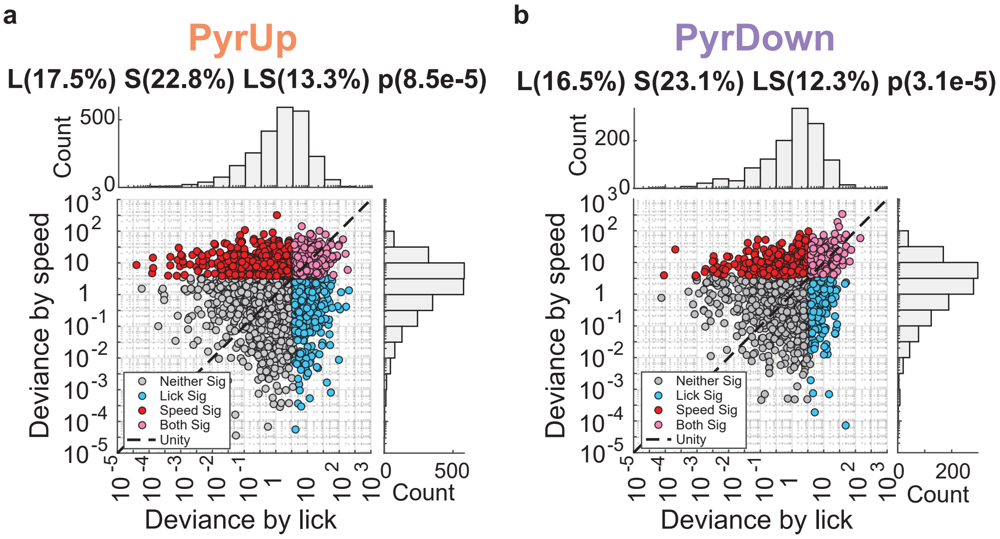

Supplementary Fig. 8: GLM analyses on PyrUp and PyrDown neurons

(a) Lick versus speed contribution to PyrUp neuronal activity. X-values represent the deviance explained by lick (see Methods). Y-values represent the deviance explained by speed. Each point represents a single neuron. Points colored in pink are significantly influenced by both lick and speed. Points colored in blue are significantly influenced by just lick. Points colored in red are significantly influenced by just speed. Points colored in grey are not significantly influenced by either lick or speed. The top panel shows the histogram of deviance by lick, and the right panel shows the histogram of deviance by speed. Title shows the percentage of neurons that are significantly influenced by just lick (L), just speed (S), and both lick and speed (LS). P-value is calculated using Wilcoxon rank-sum test between the deviance by lick and by speed.

(b) Same as in (a), for PyrDown neurons.

Supplementary Fig. 9

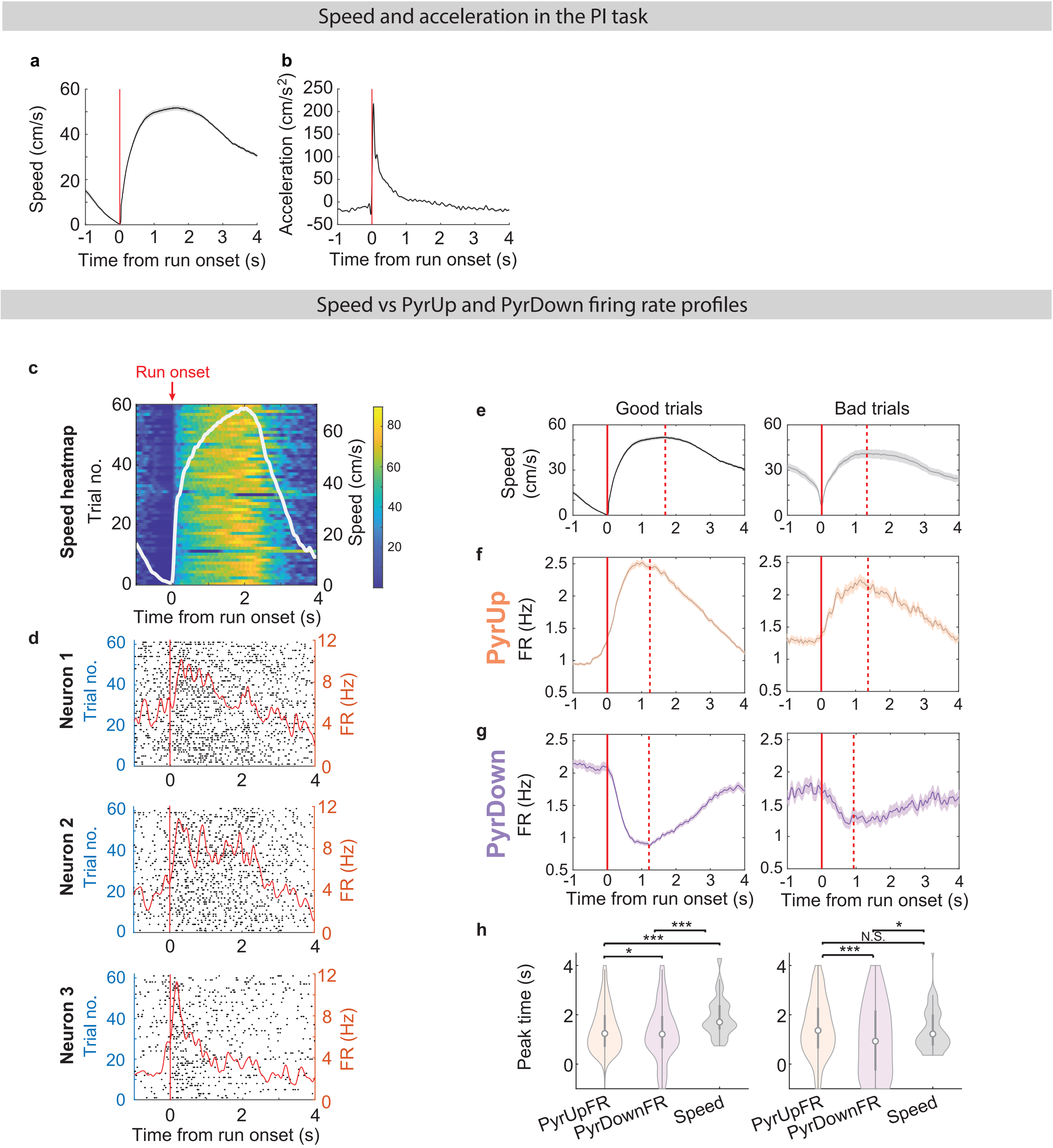

Supplementary Fig. 9: Speed and acceleration, and speed versus PyrUp/PyrDown firing rate profiles

(a) Speed profile over time averaged across recordings.

(b) Acceleration profile over time averaged across recordings.

(c-d) (c) Heatmap of speed as a function of time from run onset over trials (mean speed profile shown in white). (d) Spike raster plots of three example PyrUp neurons from the same session (mean firing rate profile over time shown in red), demonstrating that PyrUp activity reaches the peak before the running speed.

(e-g) (e) Speed profile, (f) PyrUp firing rate profile, and (g) PyrDown firing rate profile, averaged across recordings. Left: good trials; right: bad trials. Red dashed line, median peak/trough time over recordings.

(h) Peak time of PyrUp/PyrDown firing rate profiles and speed profiles. Left: good trials; right: bad trials. The peak time difference between PyrUp response and speed is not significant in bad trials.

Supplementary Fig. 10

PyrUp and PyrDown responses are specific to run onset at the trial start in the path integration task

Spontaneous run onset vs. Run onset at trial start

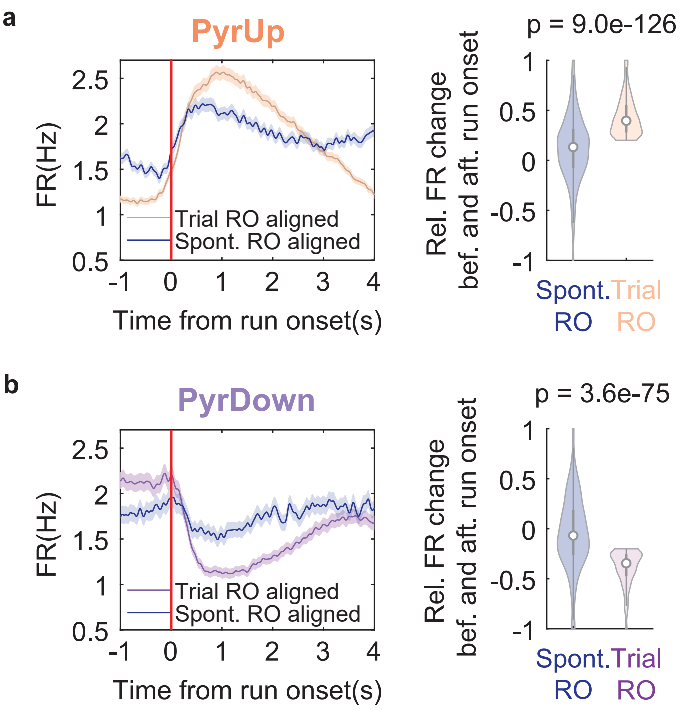

Spontaneous run onset vs. Run onset at trial start (speed matched)

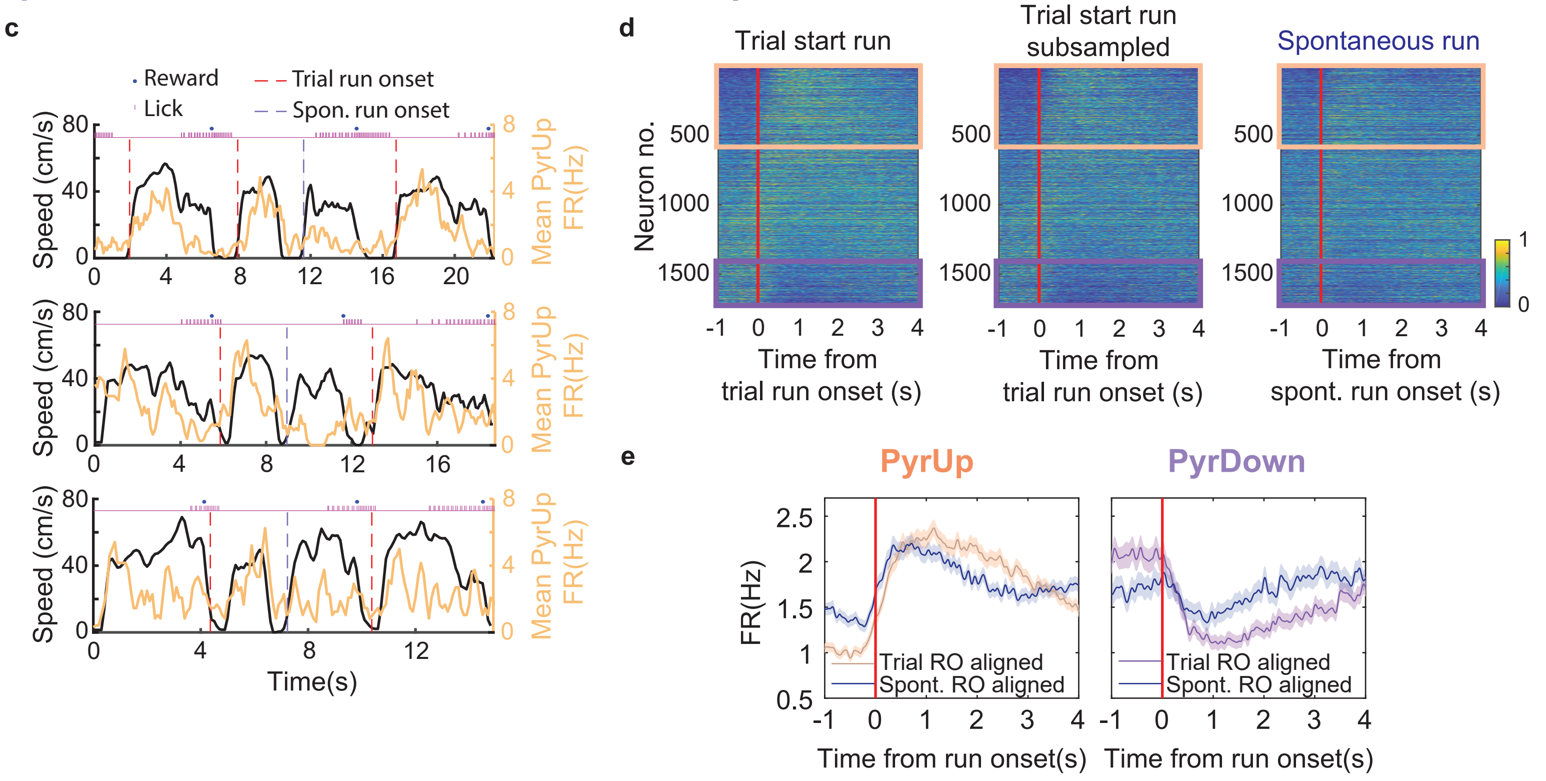

Supplementary Fig. 10: PyrUp/PyrDown responses are specific to run onset at the trial start in the PI task

(a) Left: After realigning with spontaneous run onset (SRO), the firing rate profile averaged across PyrUp neurons (dark blue) shows smaller changes than after aligning with the trial-start run onset (TRO). Recordings with at least 15 spontaneous run bouts are included in the analysis. Right: Relative firing rate change around run onset. At SRO: 0.12±0.010, at TRO: 0.44±0.0061 (960/2902 neurons).

(b) Same as in (a), for PyrDown neurons. At SRO: -0.04±0.016, at TRO: -0.39±0.008 (513/2902 neurons).

(c-e) Spontaneous run bouts speed-matched with the trial-start run bouts (see Methods). (c) Example locomotion traces (speed over time) from three representative recordings from three animals. Each example shows three trial-start runs and one speed-matched spontaneous run occurring in the middle of the second trial. Speed traces (black) are overlaid with the population-averaged PyrUp neuron activity from the same trials (orange). (d) Individual PyrUp/PyrDown neurons aligned with trial-onset (left) versus spontaneous run-onset (right, PyrUp: 582/1731 neurons, PyrDown: 325/1731 neurons). Results suggest a reduction in run-onset response during the spontaneous runs, even after subsampling the trial start runs to match the number of spontaneous runs per recording (middle). (e) Similar to (a) and (b), after selecting recordings with matching running speed between spontaneous runs and runs from the trial start.

Supplementary Fig. 11

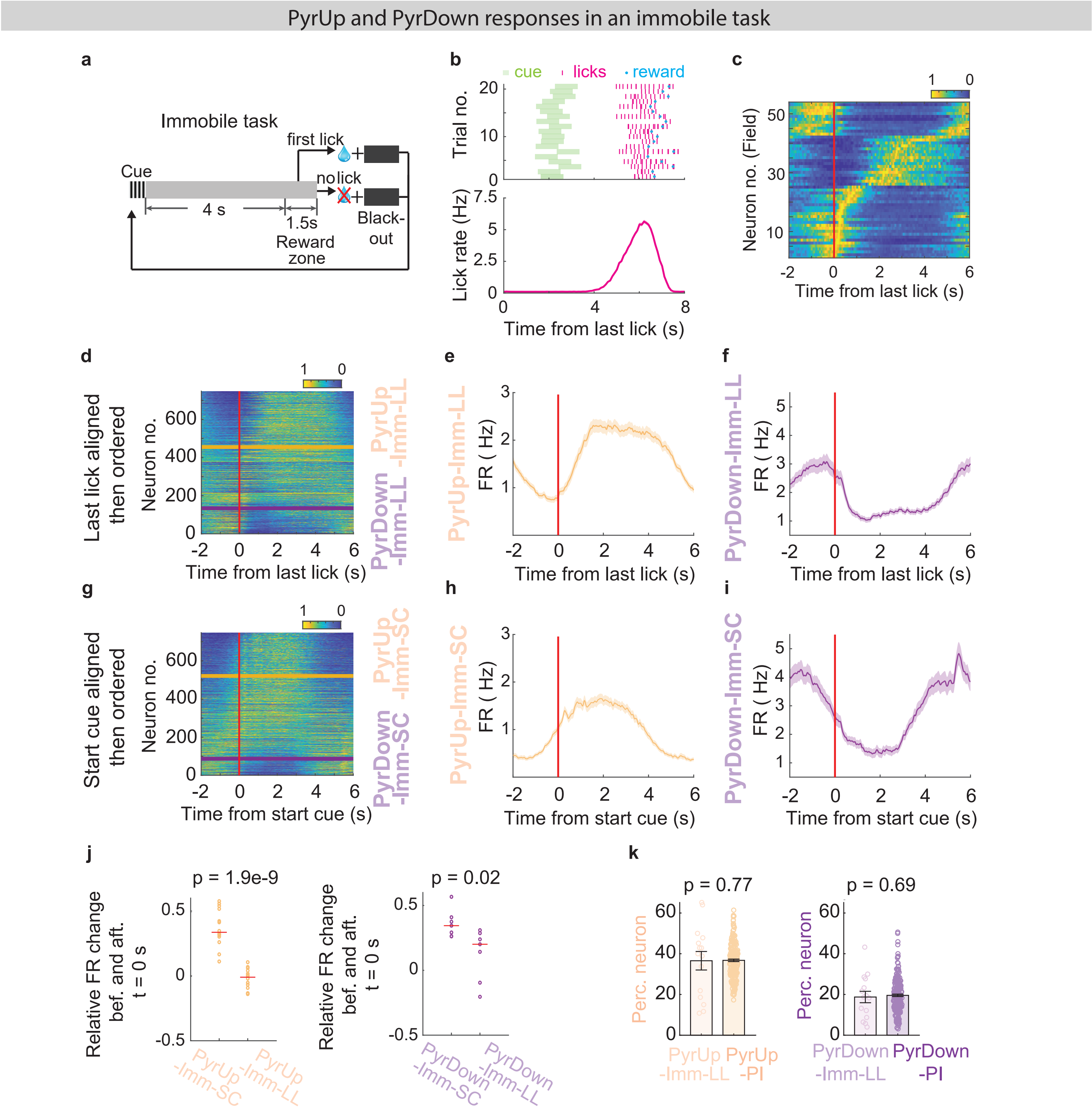

Supplementary Fig. 11: PyrUp/PyrDown responses in an immobile task
(a) Task schematic for the immobile task.
(b) Anticipatory licking in the immobile task. Top: example trials aligned with the last lick from the previous trial. Start cue: green; reward: blue; licks: magenta. Bottom: averaged lick profile excluding consummatory licks across recordings (505 trials). The anticipatory licking pattern supports that the animals were integrating time to guide their reward-seeking behavior.
(c) IGS is present after aligning the neuronal activity with the last lick of the previous trial (53/748 neurons).
(d) Normalized firing rate heatmaps of all pyramidal neurons ordered by their firing rate ratio $R = FR_{aft}/FR_{bef}$ (FR_{bef}: mean firing rate (FR) before last lick (-1.5 to -0.5s), FR_{aft}: FR after last lick (0.5 to 1.5s), 0s corresponds to the last lick). PyrUp-Imm-LL (274 neurons, $R > 3/2$) and PyrDown-Imm-LL (142 neurons, $R < 2/3$) neurons are separated by orange and purple lines, respectively.
(e) Firing rate profile averaged across all PyrUp-Imm-LL neurons.
(f) Same as in (e), for PyrDown-Imm-LL neurons.
(g-i) Same as in (d-f), aligned with the start cue instead of the last lick of the previous trials. PyrUp-Imm-SC and PyrDown-Imm-SC neurons are identified after start-cue alignment using the same criteria in (d).
(j) Relative FR change at t= 0s from the mean baseline activity (-1.5 to -0.5s), compared between start-cue aligned and last-lick aligned activity per recording. Left: PyrUp-Imm neurons; right: PyrDown-Imm neurons.
(k) Left: Percentage of PyrUp neurons identified in the immobile task (light yellow) is comparable to that identified in the PI task (orange). Right: same as left, for PyrDown neurons.

Supplementary Fig. 12

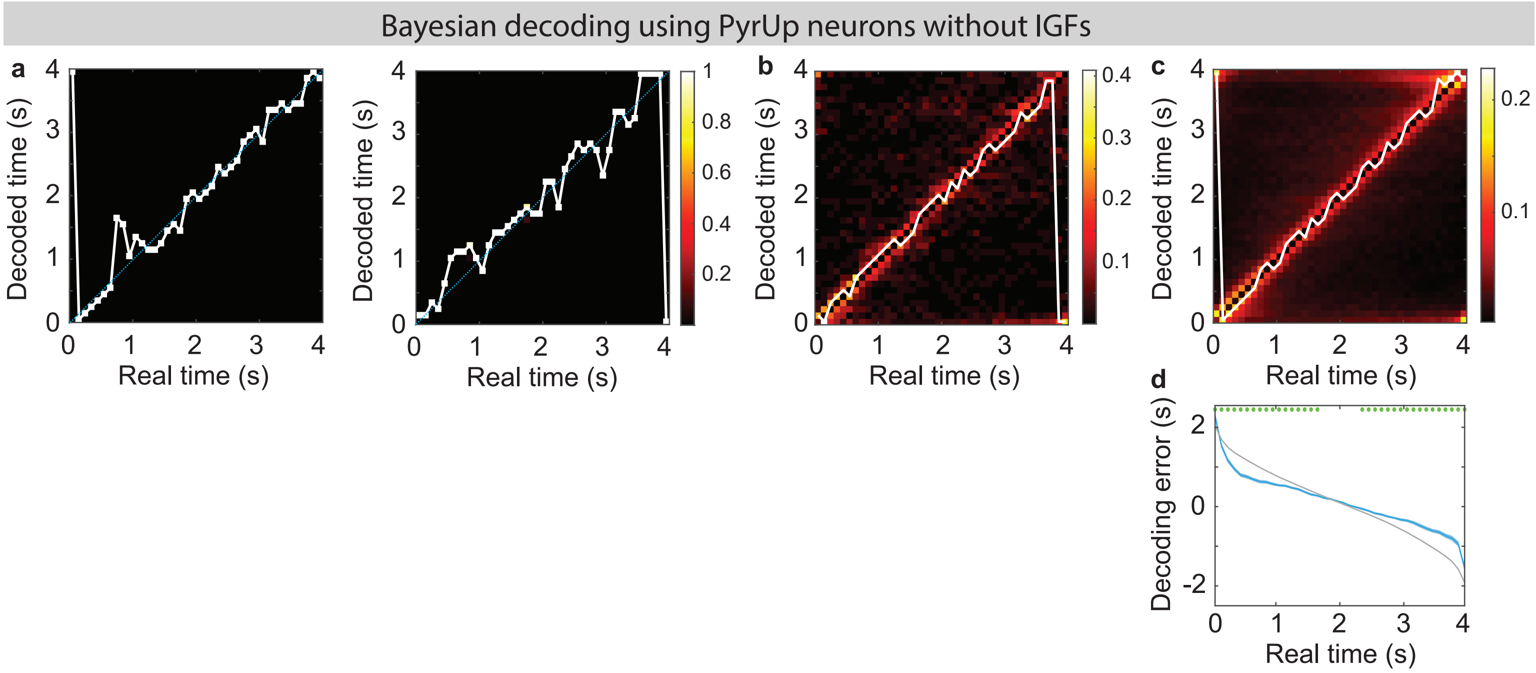

Supplementary Fig. 12: Bayesian decoding using PyrUp neurons without IGFs
(a) Decoding results of two single trials. Color bar indicates posterior probabilities. White line denotes the decoder's most confident estimation.
(b) Decoding results of a single recording session, the same session as in (a).
(c) Decoding results averaged across all recording sessions (23 animals, 70 recordings).
(d) Decoding error determined using tenfold cross-validation (blue) compared with the shuffled data (grey), averaged across all recording sessions. Green dots highlight time points where the decoding error differ significantly from that in shuffled data.

Supplementary Fig. 13

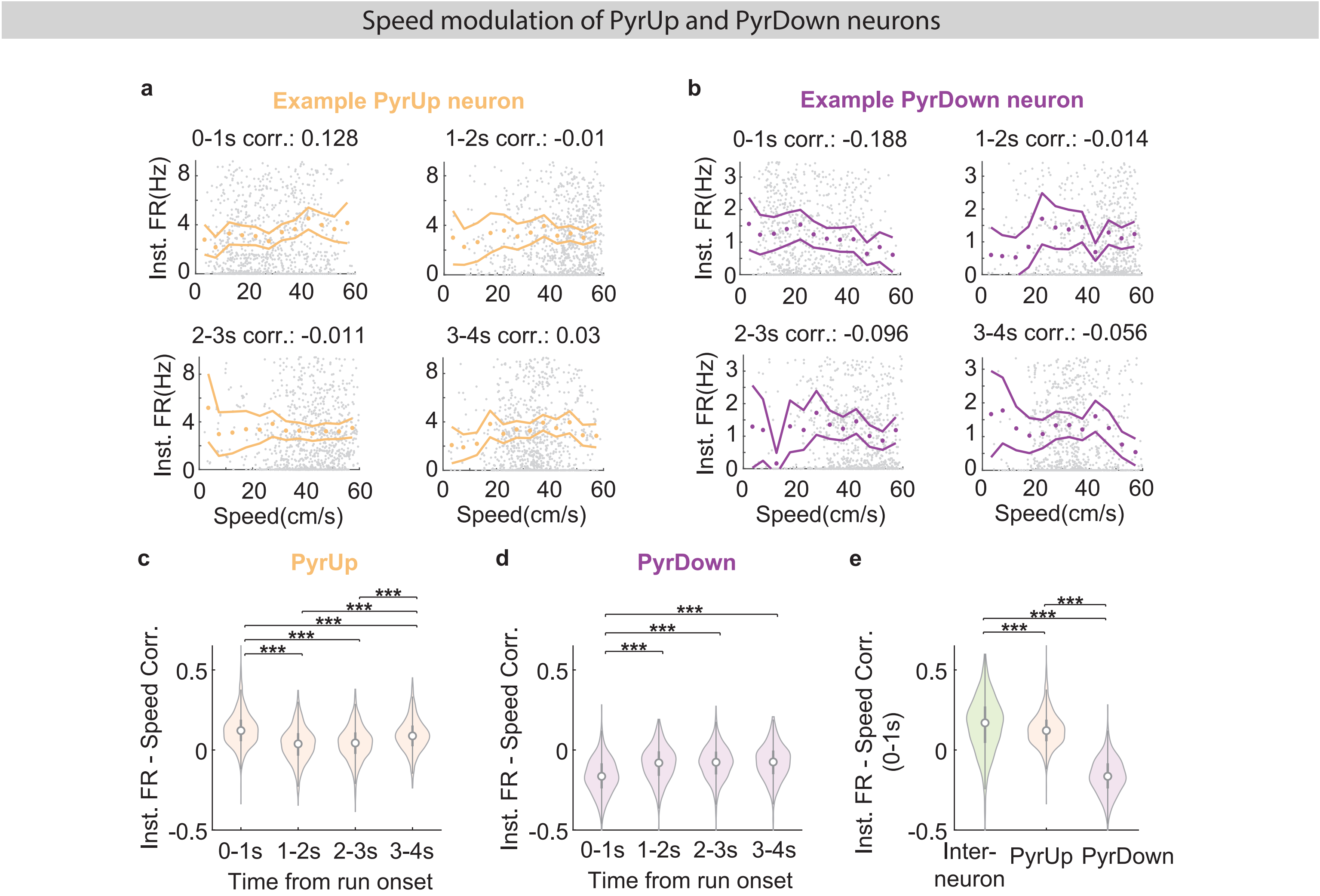

Supplementary Fig. 13: Speed modulation of PyrUp and PyrDown neurons

(a) Scatter plots of running speed versus instantaneous firing rate for an example PyrUp neuron. Individual measurements are shown as grey dots, and mean firing rates within speed bins are shown as yellow dots. Yellow lines indicate the 95% confidence interval for the mean firing rate in each bin. Plots are broken up by trial time relative to the run onset (see Methods). The correlation coefficient for each time window is displayed above each subplot.

(b) Same as in (a), for an example PyrDown neuron.

(c) Distribution of correlation coefficients between instantaneous firing rate and running speed for the entire population of PyrUp neurons, shown for sequential one-second time bins (***$p < 0.005$). Neurons with >200 spikes for each bin are selected for analysis (1028 neurons).

(d) Same as in (c), for PyrDown neurons (455 neurons).

(e) Comparison of speed correlation during the initial run phase (0-1 s after run onset) across three neural populations: interneurons (784 neurons), PyrUp neurons, and PyrDown neurons (***$p < 0.005$).

Supplementary Fig. 14

PyrUp and PyrDown neurons in the passive task

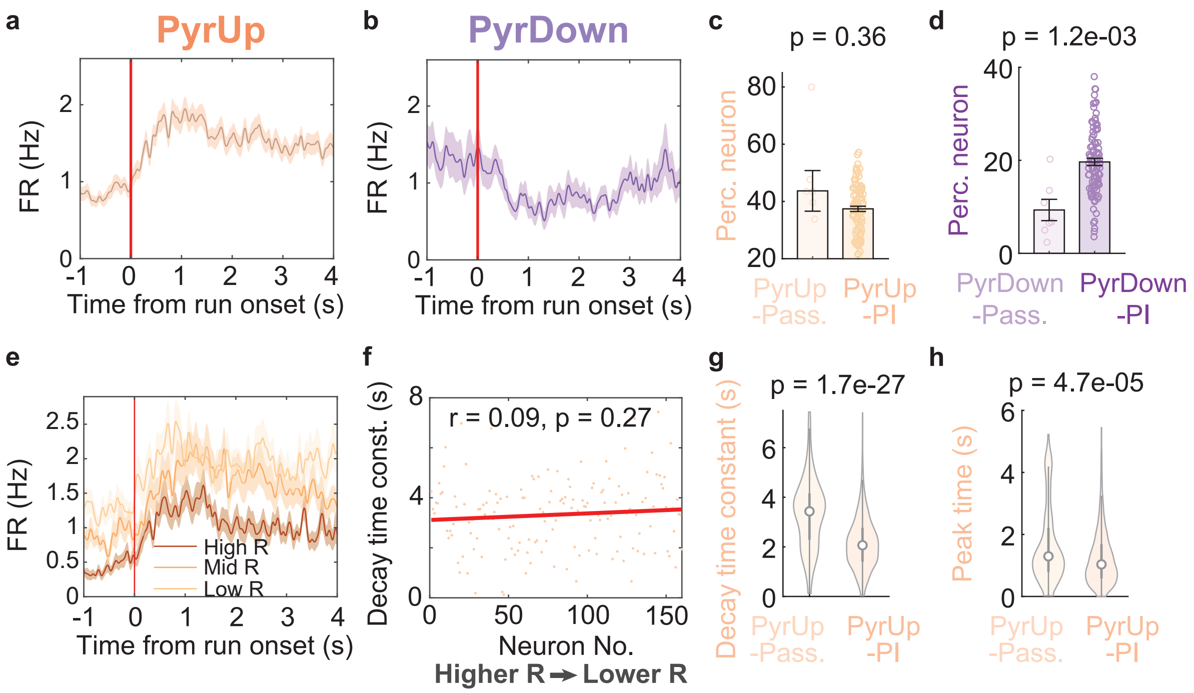

Supplementary Fig. 14: PyrUp and PyrDown neurons in the passive task

(a) The firing rate profile averaged across all PyrUp neurons. The shaded area shows the SEM.

(b) Same as in (a), for PyrDown neurons.

(c) Percentage of PyrUp neurons in the passive and PI tasks.

(d) Percentage of PyrDown neurons in the passive and PI tasks.

(e) Average firing rate profiles among three equal-sized PyrUp neuron groups based on their R values: high R, mid R, and low R (dark to light lines).

(f) R versus decay time constant for PyrUp neurons (orange dots; see Methods). The linear regression line (red) illustrates the change of decay time constants across neurons.

(g) Decay time constant of PyrUp neurons in the passive and PI tasks.

(h) Peak time of PyrUp neurons in the passive and PI tasks.

Supplementary Fig. 15

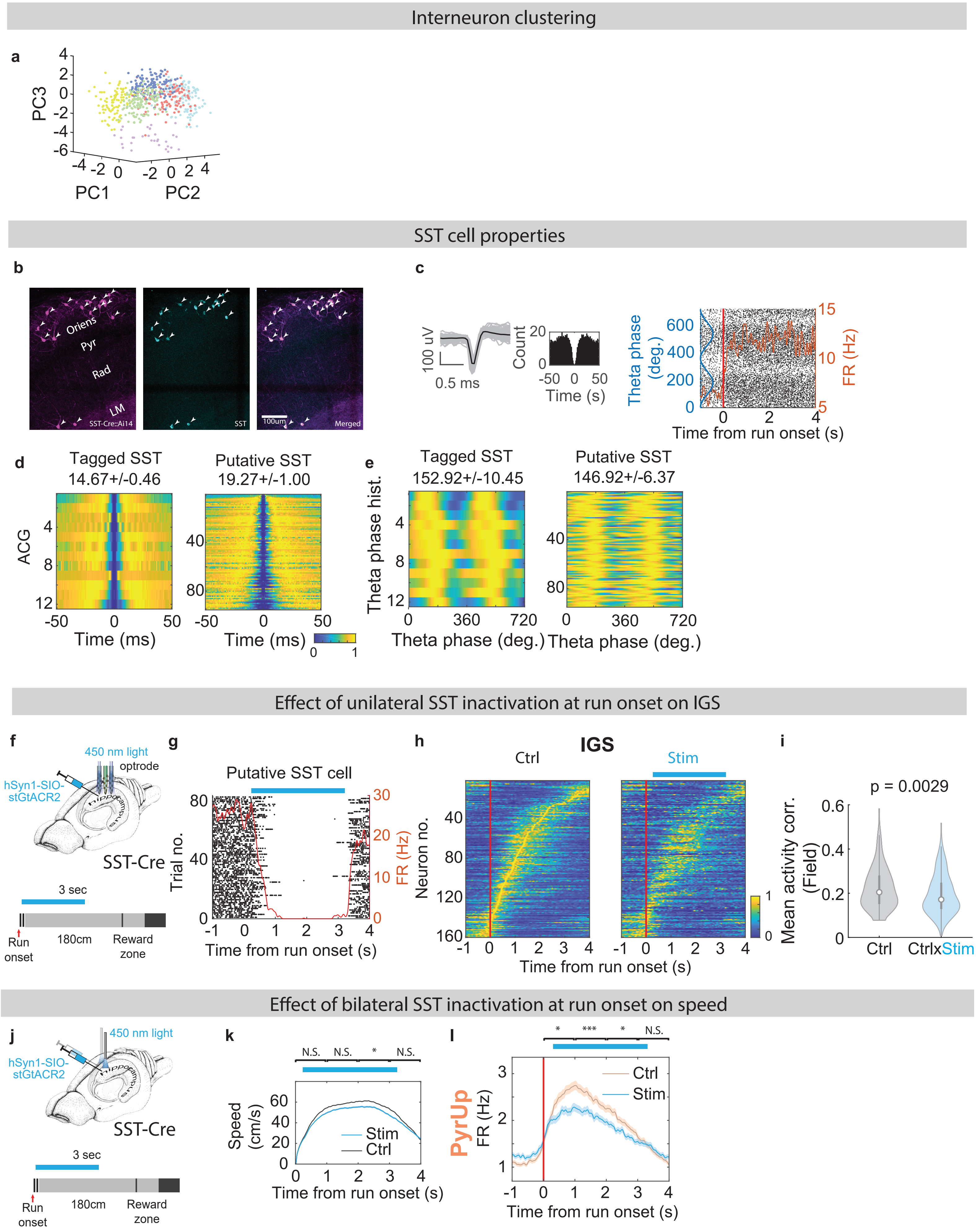

Supplementary Fig. 15: SST interneuron optogenetic tagging and inactivation at run onset

(a) A three-dimensional view of the 6 interneuron clusters in the PCA space. Each point represents one neuron.
(b) Confocal images of CA1 from an SST-Cre x Ai14 mouse. From left to right, tdTomato expression, SST immunostaining (GFP), and overlaid images. Arrows point to cells that are co-labeled for tdTomato and GFP.
(c) Spike waveform (left) and auto-correlogram (middle) of a putative SST interneuron. In the left panel, single waveforms are shown in grey and the averaged waveform is shown in black. Raster plot (right) showing spike theta phases (black dots) and firing rate as a function of time (orange) for the same cell.
(d) Auto-correlograms (ACGs) of tagged (left, as in Fig. 5b) and putative SST cells (right, as in Fig. 5d). On the top shows the mean±SEM of the ACG peak time averaged across all cells.
(e) Theta phase histograms of tagged (left) and putative SST cells (right). On the top shows the mean±SEM of the peak theta phase averaged across all cells.
(f-g) Effect of unilateral optrode-mediated SST inactivation at run onset on IGS. (f) From Fig. 5g, experimental setup[7]. (g) An example putative SST cell that is effectively inactivated by light.
(h) IGS during control (left) and stimulation trials (right).
(i) Mean activity correlation of neurons with IGFs, comparing control vs. control (grey) and control vs. stimulation trials (blue).
(j-l) Effect of bilateral SST interneuron inactivation at run onset on speed. (j) From Fig. 5l, experimental setup[7]. (k) Speed profile over time for control (grey) and stimulation trials (blue). (l) For comparison, firing rate profile averaged across PyrUp neurons from Fig. 5i (unilateral SST inactivation) is shown here. Statistics are calculated between control and stimulation trials on the mean value within each second from 0 to 4 seconds, * $p < 0.05$, ** $p < 0.01$, *** $p < 0.005$, Wilcoxon rank-sum test.

Supplementary Fig. 16

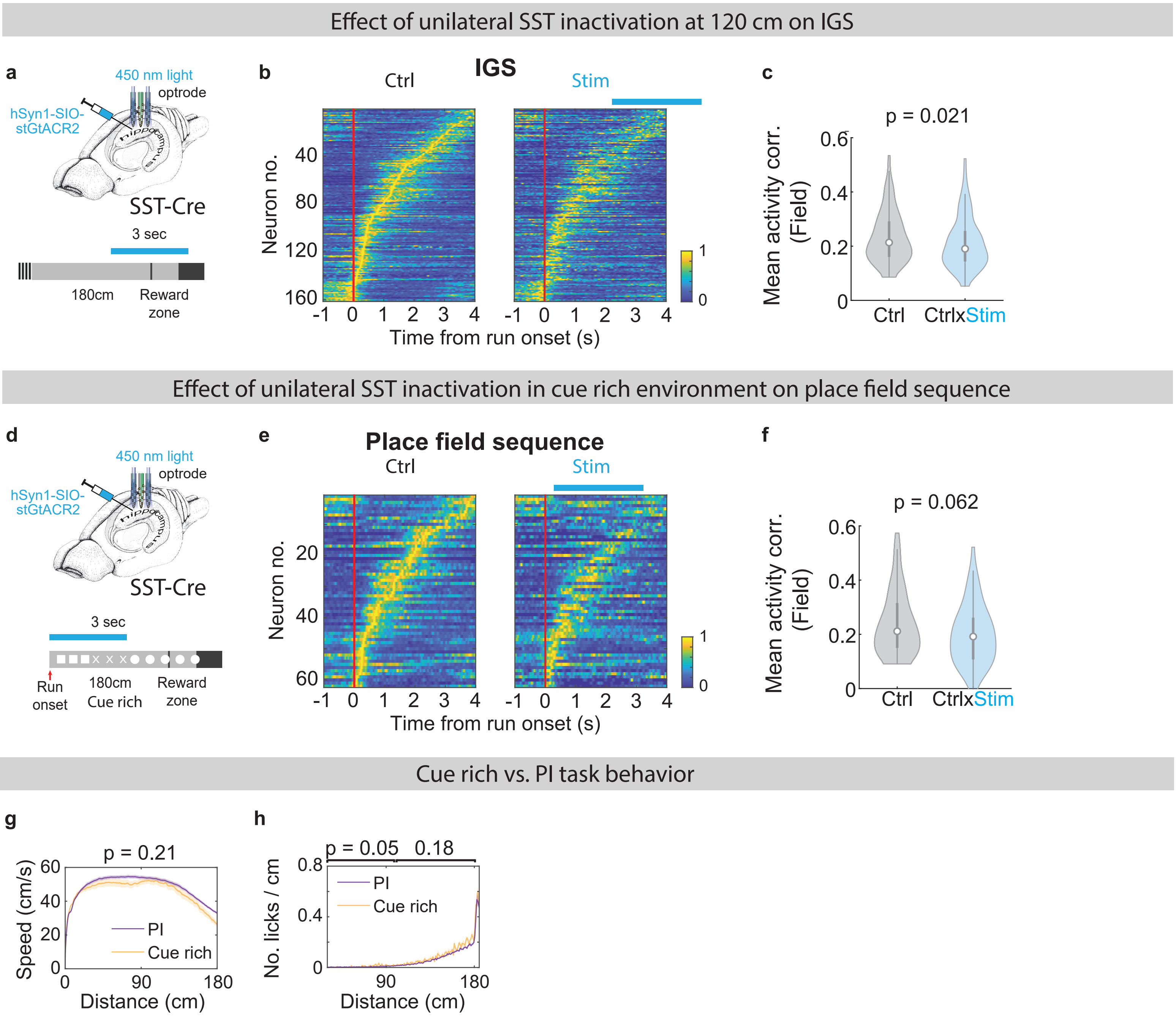

Supplementary Fig. 16: SST interneuron inactivation at 120 cm and in cue rich environment

(a-c) Effect of optrode-mediated SST inactivation at 120 cm on IGS. (a) From Fig. 6a, experimental setup[7]. (b) IGS during control (left) and stimulation trials (right). (c) Mean activity correlation of neurons with IGFs, comparing control vs. control (grey) and control vs. stimulation trials (blue).

(d-f) Effect of optrode-mediated SST inactivation in a cue-rich environment on place field sequence. (d) From Fig. 6k, experimental setup[7]. (e) Place field sequence during control (left) and stimulation trials (right). (f) Mean activity correlation of place cells, comparing control vs. control (grey) and control vs. stimulation trials.

(g-h) Behavior in cue rich environment versus the PI task. (g) Speed profile over distance, averaged across recordings. Cue rich: orange; PI: purple. The shaded area represents SEM.

(h) Licking profile over distance, averaged across recordings. P-values for time windows 0-2s and 2-4s are shown.

Supplementary Fig. 17

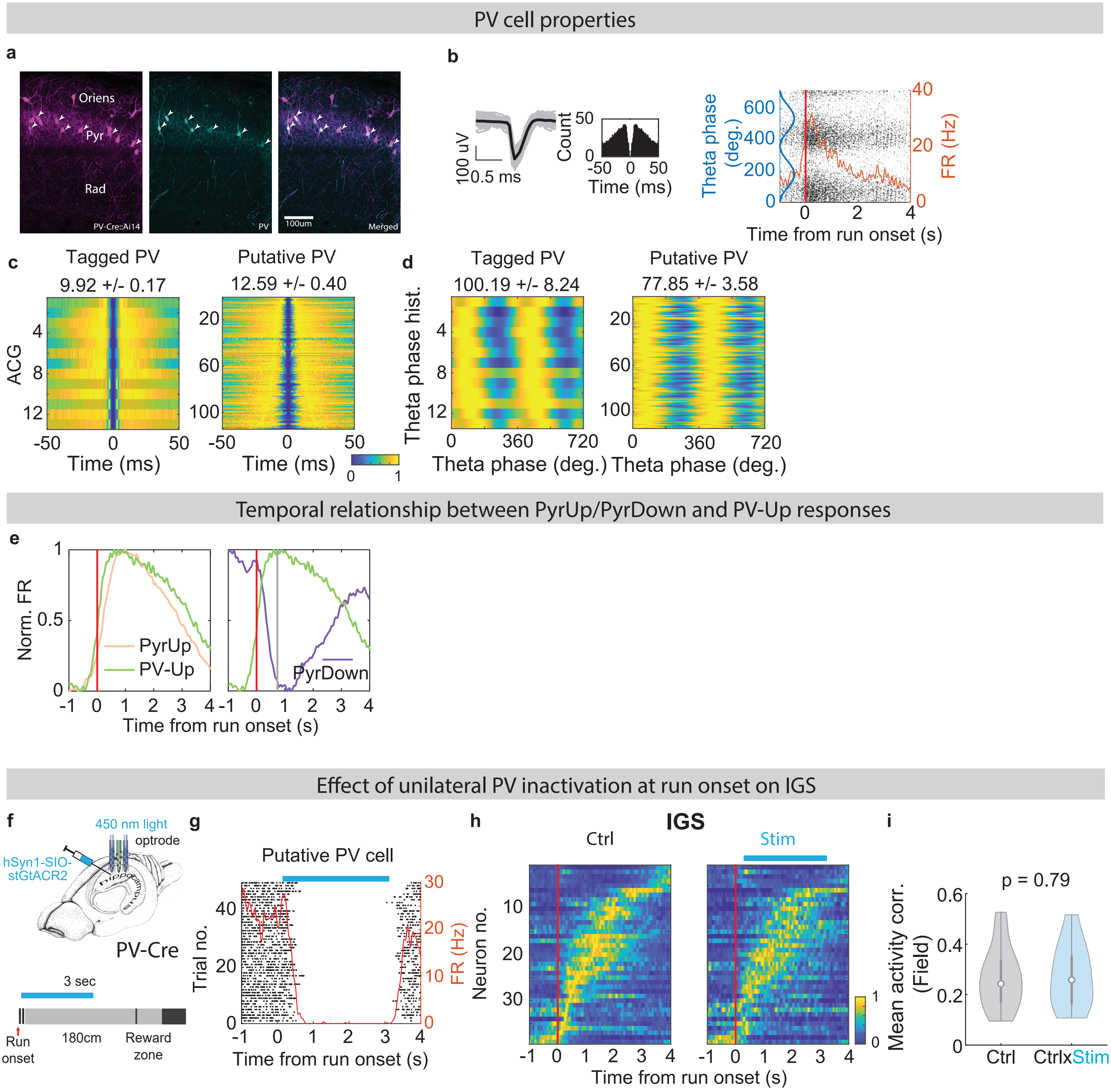

Supplementary Fig. 17: PV interneuron optogenetic tagging and inactivation at run onset
(a) Confocal images of CA1 from a PV-Cre x Ai14 mouse. From left to right, tdTomato expression, PV immunostaining (GFP), and overlaid images. Arrows point to cells that are co-labeled for tdTomato and GFP.
(b) Spike waveform (left) and auto-correlogram (middle) of a putative PV interneuron. In the left panel, single waveforms are shown in grey and the averaged waveform is shown in black. Raster plot (right) showing spike theta phases (black dots) and firing rate as a function of time (orange) for the same cell.
(c) Auto-correlograms (ACGs) of tagged (left, as in Fig. 7b) and putative PV cells (right, as in Fig. 7d). On the top shows the mean±SEM of the ACG peak time averaged across all cells.
(d) Theta phase histograms of tagged (left) and putative PV cells (right). On the top shows the mean±SEM of the peak theta phase averaged across all cells.
(e) Averaged normalized firing rate profiles of PyrUp and PV-Up neurons (left), or PyrDown and PV-Up neurons (right). The peak of PV-Up neurons' activity is denoted by the grey line (right).
(f-g) Effect of unilateral optrode-mediated PV inactivation at run onset on IGS. (f) From Fig. 7g, experimental setup[7]. (g) An example putative PV cell that is effectively inactivated by light.
(h) IGS during control (left) and stimulation trials (right).
(i) Mean activity correlation of neurons with IGFs, comparing control vs. control (grey) and control vs. stimulation trials.

Supplementary Fig. 18

Control experiments for bilateral optogenetic inactivation

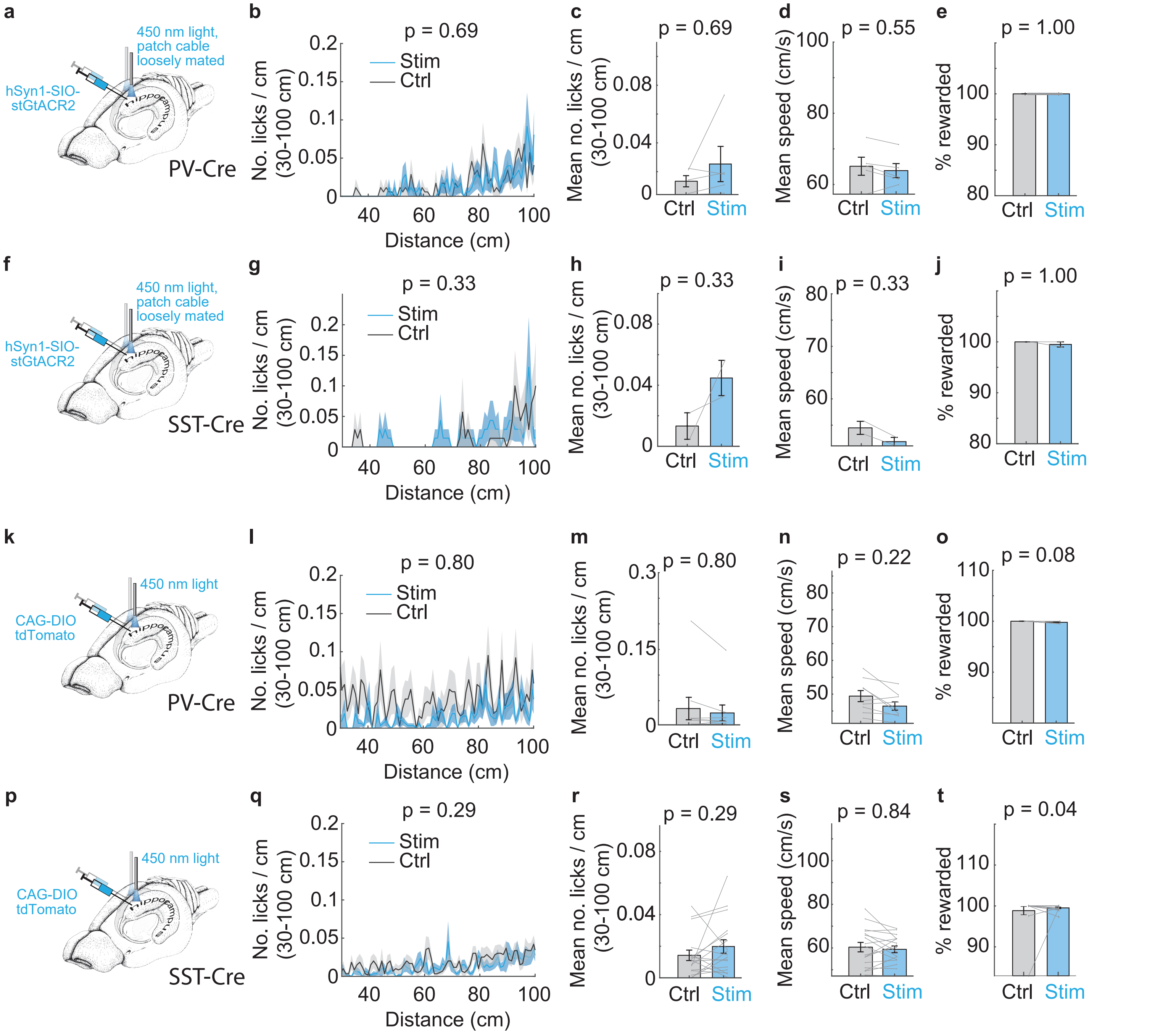

Supplementary Fig. 18: Control experiments for bilateral optogenetic inactivation
(a-e) Control experiments for optogenetic inactivation of PV interneurons (3 animals). (a) Experimental design[7]. Light delivery protocols are identical to previous experiments, but the patch cables were adjusted so they did not contact the implanted fiber optic cannulas, precluding light delivery into the brain. (b) Lick histogram for 30-100 cm for control and stimulation sessions. Reported p-value is for the mean number of licks in this same period. (c) Mean number of licks/cm calculated based on (b). (d) Mean running speed. (d) Percentage of rewarded trials.
(f-j) The same as (a-e), for control experiments for inactivation of SST interneurons (2 animals).
(k-o) The same as (a-e), for control experiments using interneurons infected with tdTomato (2 animals).
(p-t) The same as (f-j), for control experiments using interneurons infected with tdTomato (4 animals).
