# Supplementary material for: Time or distance encoding by hippocampal neurons via heterogeneous ramping rates
Source: Nat Commun. 2025 Dec 17;16:11083. doi: 10.1038/s41467-025-67038-3 (PMC12712063; doi:10.1038/s41467-025-67038-3)
Supplement: Supplementary file 2 — Reporting Summary [file 41467_2025_67038_MOESM2_ESM.pdf]

nature portfolio

Corresponding author(s): Yingxue Wang

Last updated by author(s): Nov 13, 2025

Reporting Summary

Nature Portfolio wishes to improve the reproducibility of the work that we publish. This form provides structure for consistency and transparency in reporting. For further information on Nature Portfolio policies, see our Editorial Policies and the Editorial Policy Checklist.

Statistics

For all statistical analyses, confirm that the following items are present in the figure legend, table legend, main text, or Methods section.

n/a	Confirmed	
☐	☒	The exact sample size (*n*) for each experimental group/condition, given as a discrete number and unit of measurement
☐	☒	A statement on whether measurements were taken from distinct samples or whether the same sample was measured repeatedly
☐	☒	The statistical test(s) used AND whether they are one- or two-sided<br>*Only common tests should be described solely by name; describe more complex techniques in the Methods section.*
☒	☐	A description of all covariates tested
☒	☐	A description of any assumptions or corrections, such as tests of normality and adjustment for multiple comparisons
☐	☒	A full description of the statistical parameters including central tendency (e.g. means) or other basic estimates (e.g. regression coefficient) AND variation (e.g. standard deviation) or associated estimates of uncertainty (e.g. confidence intervals)
☐	☒	For null hypothesis testing, the test statistic (e.g. *F*, *t*, *r*) with confidence intervals, effect sizes, degrees of freedom and *P* value noted<br>*Give P values as exact values whenever suitable.*
☐	☒	For Bayesian analysis, information on the choice of priors and Markov chain Monte Carlo settings
☒	☐	For hierarchical and complex designs, identification of the appropriate level for tests and full reporting of outcomes
☒	☐	Estimates of effect sizes (e.g. Cohen's *d*, Pearson's *r*), indicating how they were calculated

Our web collection on statistics for biologists contains articles on many of the points above.

Software and code

Policy information about availability of computer code

Data collection	For behavioral data acquisition, we used custom Arduino (https://www.arduino.cc/) and Matlab code. For electrophysiological recordings, we used AmpliRec software (http://www.amplipex.com/downloads/). For virtual-reality environments, we used custom Unity (https://unity.com/) code.
Data analysis	For confocal image analysis, we used ImageJ (https://fiji.sc/). For spike sorting, we used open-source software Kilosort (https://github.com/jamesjun/Kilosort2), Klusters, Neuroscope, and NDManager(https://klustakwik.sourceforge.net/). For data analysis, we used Matlab to write custom code (https://www.mathworks.com/products/matlab.html)

For manuscripts utilizing custom algorithms or software that are central to the research but not yet described in published literature, software must be made available to editors and reviewers. We strongly encourage code deposition in a community repository (e.g. GitHub). See the Nature Portfolio guidelines for submitting code & software for further information.

Data

Policy information about availability of data

All manuscripts must include a data availability statement. This statement should provide the following information, where applicable:

- Accession codes, unique identifiers, or web links for publicly available datasets
- A description of any restrictions on data availability
- For clinical datasets or third party data, please ensure that the statement adheres to our policy

Data corresponding to all main figures are available at the following open source repository: https://doi.org/10.17617/3.I2QI6Q. Source data are provided with this

paper.

Custom scripts were written in MATLAB 2023b. Code related to this paper is available at GitHub (https://github.com/the-wang-lab/Code-Heldman-NC-2025).

Research involving human participants, their data, or biological material

Policy information about studies with human participants or human data. See also policy information about sex, gender (identity/presentation), and sexual orientation and race, ethnicity and racism.

Reporting on sex and gender	*Use the terms sex (biological attribute) and gender (shaped by social and cultural circumstances) carefully in order to avoid confusing both terms. Indicate if findings apply to only one sex or gender; describe whether sex and gender were considered in study design; whether sex and/or gender was determined based on self-reporting or assigned and methods used. Provide in the source data disaggregated sex and gender data, where this information has been collected, and if consent has been obtained for sharing of individual-level data; provide overall numbers in this Reporting Summary. Please state if this information has not been collected. Report sex- and gender-based analyses where performed, justify reasons for lack of sex- and gender-based analysis.*
Reporting on race, ethnicity, or other socially relevant groupings	*Please specify the socially constructed or socially relevant categorization variable(s) used in your manuscript and explain why they were used. Please note that such variables should not be used as proxies for other socially constructed/relevant variables (for example, race or ethnicity should not be used as a proxy for socioeconomic status). Provide clear definitions of the relevant terms used, how they were provided (by the participants/respondents, the researchers, or third parties), and the method(s) used to classify people into the different categories (e.g. self-report, census or administrative data, social media data, etc.) Please provide details about how you controlled for confounding variables in your analyses.*
Population characteristics	*Describe the covariate-relevant population characteristics of the human research participants (e.g. age, genotypic information, past and current diagnosis and treatment categories). If you filled out the behavioural & social sciences study design questions and have nothing to add here, write "See above."*
Recruitment	*Describe how participants were recruited. Outline any potential self-selection bias or other biases that may be present and how these are likely to impact results.*
Ethics oversight	*Identify the organization(s) that approved the study protocol.*

Note that full information on the approval of the study protocol must also be provided in the manuscript.

Field-specific reporting

Please select the one below that is the best fit for your research. If you are not sure, read the appropriate sections before making your selection.

[x] Life sciences [ ] Behavioural & social sciences [ ] Ecological, evolutionary & environmental sciences

For a reference copy of the document with all sections, see nature.com/documents/nr-reporting-summary-flat.pdf

Life sciences study design

All studies must disclose on these points even when the disclosure is negative.

Sample size	For electrophysiological recordings in the active task, we have included the data from twenty eight animals. For electrophysiological recordings in the passive task, three animals were included. For electrophysiological recordings in the immobile task, five animals were included. For optogenetic tagging of SST interneurons, three animals were included. For electrophysiological recordings with optogentic inactivation of SST interneurons, six animals were included. For bilateral optogenetic inactivation of SST interneurons, six animals were included. For electrophysiological recordings with optogentic inactivation of the SST interneurons in the cue-rich environment, three animals were included. For optogenetic tagging of PV interneurons, seven animals were included. For electrophysiological recordings with optogentic inactivation of PV interneurons, three animals were included. For bilateral optogenetic inactivation of PV interneurons, four animals were included.
Data exclusions	Analysis of brain state change, we excluded recordings that had movement artifacts in the delta and theta frequency bands. For electrophysiological recordings with optogentic activation of PV interneurons, we have excluded recordings where there was a clear stimulation artifact during the stimulation starting at 120 cm during the cue-constant segment. For bilateral optogenetic experiments, we excluded recordings where there was clear licking artifacts in the control sessions, and where the running speed in the control session is less than 50 cm/s. Also, for the bilateral optogenetic experiments, we excluded the data from one animal whose fiber location in one hemisphere was misplaced based on histological inspection.
Replication	We made sure that the results were reproducible both within and across subjects. For the bilateral optogenetic stimulation experiments, two investigators performed the experiments independently and replicated the same results. For data analysis, we typically used more than one method to analyze the data to make sure the results were robust.
Randomization	N/A. Comparisons were done within subject. In all the optogenetic experiments, we compared control trials with stimulation trials within the same session. The stimulation types (stimulating at running onset, reward location or at 120 cm during the cue-constant segment) were randomized. Control sessions where the optogenetics patch cables were only loosely mated with the implanted fiber optic cannulas to prevent light from propagating into CA1, were done in the same cohort of animals as those used for optogenetic activation and inactivation

experiments.

Blinding

During data analysis, we automatically ran the same code through all the experimental conditions. Therefore, the investigators were blinded to the group allocation.

Reporting for specific materials, systems and methods

We require information from authors about some types of materials, experimental systems and methods used in many studies. Here, indicate whether each material, system or method listed is relevant to your study. If you are not sure if a list item applies to your research, read the appropriate section before selecting a response.

Materials & experimental systems

n/a	Involved in the study
☐	☒ Antibodies
☒	☐ Eukaryotic cell lines
☒	☐ Palaeontology and archaeology
☐	☒ Animals and other organisms
☒	☐ Clinical data
☒	☐ Dual use research of concern
☒	☐ Plants

Methods

n/a	Involved in the study
☒	☐ ChIP-seq
☒	☐ Flow cytometry
☒	☐ MRI-based neuroimaging

Antibodies

Antibodies used

guinea pig polyclonal anti-Parvalbumin
Swant
Cat# GP72,RRID:AB_2665495

Alexa Fluor 488 AffiniPure Donkey Anti-Guinea Pig IgG (H+L)
Jackson ImmunoResearch Laboratories
Cat# 706-545-148, RRID: AB_2340472

Anti-Somatostatin Antibody, clone YC7
Millipore
Cat# MAB354, RRID:AB_2255365

Alexa Fluor 488 AffiniPure Donkey Anti-Rat IgG (H+L)
Jackson ImmunoResearch Laboratories
Cat# 712-545-153, RRID: AB_2340684

Validation

For anti-Parvalbumin antibody, from the product description: "The antibody specifically localizes parvalbumin using free-floating or mounted sections of brain, kidney and muscles of probably all vertebrates The antiserum does not stain the brain of parvalbumin-KO mice.
References:
1. *Filice F., Celio, M.R., Szabolcsi V. (2017) JCN, in press"

For anti-Somatostatin Antibody, from the product description: "Synthetic peptide corresponding to amino acids 1-14 of cyclic somatostatin conjugated to bovine thyroglobulin using carbodiimide."

Animals and other research organisms

Policy information about [studies involving animals](); [ARRIVE guidelines]() recommended for reporting animal research, and [Sex and Gender in Research]()

Laboratory animals

Mice, age > 8 weeks, We used three mouse lines: C57Bl/6J (JAX #000664), PV-IRES-Cre (JAX #017320) (Hippenmeyer et al., 2005), SST-IRES-Cre (JAX #013044) (Taniguchi et al., 2011), and Ai14 (JAX # 007914) (Madisen et al., 2012).

Wild animals

No wild animals were used in this study.

Reporting on sex

This study was based on both male and female mice. Male mice were preferentially used in the running tasks because they were found to exhibit more consistent running behavior.

Field-collected samples

No field-collected samples were used in this study.

Ethics oversight

All procedures were in accordance with protocols approved by the Institutional Animal Care and Use Committee at Max Planck Florida Institute for Neuroscience.

Note that full information on the approval of the study protocol must also be provided in the manuscript.

Plants

Seed stocks	*Report on the source of all seed stocks or other plant material used. If applicable, state the seed stock centre and catalogue number. If plant specimens were collected from the field, describe the collection location, date and sampling procedures.*
Novel plant genotypes	*Describe the methods by which all novel plant genotypes were produced. This includes those generated by transgenic approaches, gene editing, chemical/radiation-based mutagenesis and hybridization. For transgenic lines, describe the transformation method, the number of independent lines analyzed and the generation upon which experiments were performed. For gene-edited lines, describe the editor used, the endogenous sequence targeted for editing, the targeting guide RNA sequence (if applicable) and how the editor was applied.*
Authentication	*Describe any authentication procedures for each seed stock used or novel genotype generated. Describe any experiments used to assess the effect of a mutation and, where applicable, how potential secondary effects (e.g. second site T-DNA insertions, mosiacism, off-target gene editing) were examined.*
